# Burnout as a predictor of depression: a cross-sectional study of the sociodemographic and clinical predictors of depression amongst nurses in Cameroon

**DOI:** 10.1186/s12912-019-0377-4

**Published:** 2019-11-01

**Authors:** Clarence Mbanga, Haman Makebe, Divine Tim, Steve Fonkou, Louise Toukam, Tsi Njim

**Affiliations:** 1 Mankon Sub-Divisional Hospital, Bamenda, Cameroon; 2 Liverpool School of Tropical Medicine, Pembroke Place, Liverpool, L3 5QA UK; 3 Clinical Research Education Networking and Consultancy, Douala, Cameroon; 4 Health and Human (2HD) Research Network, Douala, Cameroon; 5 Baptist Hospital Mutengene, Mutengene, Cameroon; 6 Faculty of Health Sciences, University of Buea, Buea, Cameroon; 7 Health Education and Research Organization (HERO), Buea, Cameroon

**Keywords:** Burnout syndrome, Nurses, Depression, Cameroon, Oldenburg burnout inventory, Patient health Questionnaire-9

## Abstract

**Background:**

Depression is a debilitating mental health condition which affects an estimated 350 million people worldwide annually. Nurses are twice as likely to suffer from depression than professionals in other professions. This leads to a considerable loss of efficiency and productivity. We sought to determine the prevalence and predictors of depression among nurses in Cameroon.

**Methods:**

Cross-sectional analysis carried out over 6 months (January – June 2018) using nurses from public and private healthcare institutions sampled consecutively in the two English-speaking regions (North west and South west regions) of Cameroon. The nurses were handed a structured, printed, self-administered questionnaire to fill and hand in at their earliest convenience. Depression and burnout were assessed using the Patient Health Questionnaire – 9 and the Oldenburg Burnout Inventory respectively.

**Results:**

A total of 143 nurses were recruited (mean age: 29.75 ± 6.55 years; age range: 20–55 years, 32.87% male). The overall prevalence of depression was 62.24%. Independent predictors of depression after multivariable analysis were: Number of night shifts a week (adjusted odds ratio: 1.58; *p* value: 0.045, 95% CI; 1.01, 2.48) and Total Oldenburg Burnout Inventory score (adjusted odds ratio: 1.21, p value: 0.001; 95% CI; 1.08, 1.35). Recreational drug use was also found to perfectly predict the outcome – depression.

**Conclusion:**

Depression is highly prevalent among nurses in the English-speaking regions of Cameroon. Accurate predictors could prove vital for early detection and management of affected individuals. Predictors presented herein require further investigation via multicentric nationwide studies, to obtain more generalizable results.

## Background

Depression is a major cause of disability worldwide as individuals with this mental health condition usually have low moods, disturbed sleep, negative feelings about life in general and anhedonia; which could lead to decrease efficiency [1–3]. In the United states, it is reported that due to loss of efficiency and productivity in nurses with depression; there is an incurred loss of about 14,339 united states dollars (USD) per nurse; which leads to an estimated national productivity loss of approximately 22.7 billion USD per year [4, 5]. About 350 million people suffer from depression worldwide annually with nurses being twice as likely to suffer from depression than professionals in other fields [1, 3, 4]. Some determinants of depression in nurses are: being married, increased age, longer durations of employment in the nursing profession, a high patient turnover and the presence of burnout syndrome [4]. Other determinants which have been shown to have an association with depression in other populations include: female sex, history of chronic disease, level of studies, occurrence of a major life event (in medical students) [6, 7]; alcohol abuse (in college students) [8]; the presence of problems in social relationships and low income levels in youths and adults [9, 10].

Burnout syndrome is a psychological state of emotional exhaustion and disengagement from work due to prolonged exposure to work environment stressors [11, 12]. It is usually described as a reaction to severe stress associated with individuals in “helping professions” [13]. There is a high workload in the health sector in Cameroon, as it is one of the 57 countries described to have a “critical shortage” of healthcare workers [14], with an average health personnel density of 1.3:1000 (below the WHO recommended value of 2.5:1000); and a nurses’ density of 0.67 per 1000 [15]. Consequently, there is an increase in patient turnover and a higher workload. As such, nurses in the country are likely to experience burnout syndrome which is possibly a predictor of depression. Some of the symptoms of burnout overlap with those of depression. These overlapping symptoms include extreme exhaustion, depressed moods and reduced performance [13]. As such, there is usually great difficulty in differentiating the two constructs. However, there are symptoms which are specific to burnout syndrome as the negative thoughts an individual may experience are exclusively work-related [13]. An individual with negative ideas about their profession will therefore not necessarily have negative thoughts about life in general.

In this setting where there is usually a lack of mental health service for the general population and for health personnel, we sought to determine the prevalence of depression amongst nurses in the two English-speaking regions of the country and to determine if burnout is a predictor of depression. This study will add to the growing cartography of mental health issues amongst nurses in Cameroon and will raise awareness on the need for the investigation of appropriate preventive measures regarding mental health conditions in the nursing population of Cameroon.

## Methods

### Population and design

This was a cross-sectional analysis carried out over a period of 6 months (January – June 2018) in the two English-speaking regions (North west and South west regions) of Cameroon. The study design was adapted from a series of papers in a project used to assess burnout and depression amongst healthcare personnel and students in Cameroon [7, 16–20]. The predictors of burnout in this population has been published in a previous paper [17]. Ethics approval was obtained from the institutional review board of the Cameroon Baptist Convention. Nurses from public and private healthcare institutions were selected by a consecutive sampling from the Regional Hospital Bamenda, Regional Hospital Limbe, Regional Hospital Buea, Mutengene Baptist Hospital and Mbingo Baptist Hospital. These hospitals were selected as they were the major functioning hospitals during the ongoing sociopolitical crises in these regions. Nurses working in these institutions at all levels of healthcare (primary, secondary and tertiary) were approached and recruited after a written informed consent was obtained. The nurses were handed a structured and piloted questionnaire to fill and hand in at their earliest convenience. A total of 143 nurses were approached and we received responses from all of them [17].

### Study instrument

Data was collected using a printed self-administered structured questionnaire with three sections.

The first section collected socio-demographic information: age; sex; marital status; hospital of practice (public or private); number of hours spent at work; predominance of shifts (night or day); presence of problems in social relationships; monthly income and the perception of sustainability on monthly income; alcohol use and history of any chronic diseases [4, 8, 21].

The second section assessed burnout syndrome using the Oldenburg Burnout Inventory (OLBI), which is a self-administered tool consisting of 16 items phrased both in the positive and negative directions. It has two main components which include exhaustion (eight items) and disengagement (eight items). Some of the items included in the exhaustion component were: “During my work, I often feel emotionally drained”; “After working, I have enough energy for my leisure activities”; “After my work, I usually feel worn out and weary”; “Usually, I can manage the amount of my work well” and “When I work, I usually feel energized”. Some of the items included in the disengagement component were: “Lately, I tend to think less at work and do my job almost mechanically”; “I find my work to be a positive challenge”; “Over time, one can become disconnected from this type of work”; “Sometimes I feel sickened by my work tasks” and “This is the only type of work that I can imagine myself doing“ [11]. The respondents answered the questions according to their perceived frequency of occurrence on a Likert-scale of one to four, ranging from strongly agree to strongly disagree. A participant could therefore have a maximum score of 64 and a minimum score of 4. Previous studies have assessed the use of the OLBI amongst medical and nursing students in Cameroon. These studies showed that the OLBI had a good reliability in these populations with an alpha Cronbach statistic of 0.74 and 0.60 in medical and nursing students respectively [7, 19].

The third section assessed depression using the reliable and valid 9-item self-administered Patient Health Questionnaire (PHQ-9) [22] which has a sensitivity of 88% and a specificity of 88% for the diagnosis of major depression when a PHQ-9 score ≥ 10 is used [23].

### Data management and statistical analysis

Data entry was done using EPI Info version 7.0 (CDC, Atlanta) software for Microsoft windows. Data entry was double-checked to minimize errors. Data analysis was done using Epi info 7.0 software for Microsoft windows and STATA version 15.0.

A provisional diagnosis of depression was made if the PHQ-9 score was > 4. The severity of depression was determined using the following classification: mild: 5–9; moderate: 10–14; moderately severe: 15–19; and severe: 20–27 [23]. The prevalence of depression amongst participants was calculated in these categories.

The two subscales of the OLBI (emotional exhaustion and disengagement) were used to produce an overall score for burnout syndrome for each participant. These subscales were added up, after reversing the score for negatively phrased questions (1 = 4, 2 = 3, 3 = 2, 4 = 1). Higher scores therefore indicated higher levels of exhaustion and disengagement, hence burnout.

Univariable analysis was performed using the Fishers exact test with categorical sociodemographic and clinical variables as predictors and depression as the outcome. For continuous variables, the student t-test was used. Significance was set at 5%. To determine the independent predictors of depression amongst the nurses, a multivariable analysis was performed where variables which were significant in the univariable analysis were inputted into a logistic regression model. The final variables that were significant in this multivariable logistic regression model were classed as independent predictors of depression.

## Results

### Sociodemographic characteristics

A total of 143 nurses were recruited. The mean age of the participants was 29.75 ± 6.55 years (age range; 20–55 years), with 47 (32.87%) being male. Summaries of the socio-demographic characteristics are shown in Tables 1 and 2.

**Table 1:** Categorical variables showing the sociodemographic characteristics of 143 nurses working in the English-speaking regions of Cameroon assessed for depression from January – June 2018

Variable		Total	
		N	%
Hospital (*n* = 132)	State-owned	90	68.18
	Private sector	42	31.82
Gender (*n* = 143)	Male	47	32.87
	Female	96	67.13
Marital status (*n* = 142)	Single	73	51.41
	Married	69	48.59
Personal relationship (*n* = 127) ^a^	Yes	78	61.42
	No	49	38.58
Difficulties in personal relationship (*n* = 118)	Yes	26	22.03
	No	92	77.97
Majority of shifts (*n* = 129)	Day	99	76.74
	Night	30	23.26
Regret career choice (*n* = 136)	Yes	23	16.91
	No	113	83.09
Occurrence of life changing crises in last 6 months (*n* = 139) ^b^	Yes	57	41.01
	No	82	58.99
Presence of chronic illness (*n* = 140) ^c^	Yes	17	12.14
	No	123	87.86
Alcohol consumption (*n* = 141)	Yes	71	50.35
	No	70	49.65
Recreational drug use (*n* = 142) ^d^	Yes	7	4.93
	No	135	95.07
Sufficient monthly income (*n* = 133)	Yes	13	9.77
	No	120	90.23

^a^Personal relationship was defined as close connections between two people formed by emotional and sexual interactions; ^b^Life changing crises defined as loss of a loved one, physical or sexual trauma and conditions of emotional or social instability ^c^Chronic illnesses included: Asthma, chronic pelvic pain, diabetes mellitus, gastroesophageal reflux disease, chronic peptic ulcer disease, migraines, cerebral lesions and paralysis; ^d^recreational drugs included: marijuana and tramadol

**Table 2:** Continuous variables showing the sociodemographic characteristics of 143 nurses working in the English-speaking regions of Cameroon assessed for depression from January – June 2018

Variable	Number of observations	Total sample			
		Mean	SD	Min	Max
Age	108	29.75	6.55	20	55
Number of hours at work a week	127	11.50	10.80	5	90
Monthly income in USD	58	125.15	75.79	0	341.59
Number of night shifts a week	101	2.19	1.55	0	8
Quantity of alcohol consumed a week	41	1.83	1.33	0.5	6.5
Total PHQ-9 score	143	6.78	4.93	0	22
Total OLBI score	143	38.36	5.68	25	52

*USD* United states dollars, *GPA* Cumulative grade point average, *OLBI* Oldenburg burnout inventory

### Prevalence of depression

The overall prevalence of depression (PHQ-9 > 4) in the study population was 62.24% (89/143). Forty-six participants (32.17%) had mild depression (PHQ-9; 5–9), 32 (22.38%) had moderate depression (PHQ-9; 10–14), 9 (6.29%) had moderately severe depression (PHQ-9; 15–19), and 2 (1.40%) participants had severe depression (PHQ-9; 20–27) (Tables 3). Most of the participants (60, 55.56%) admitted finding it somewhat difficult in dealing with the symptoms of depression they experienced (Table 4).

**Table 3:** Categorization of prevalence of depression among 143 nurses working in the English-speaking regions of Cameroon assessed for depression from January – June 2018

Category of depression	N	%
None	54	37.76
Mild depression	46	32.17
Moderate depression	32	22.38
Moderately severe depression	9	6.29
Severe depression	2	1.40
Overall depression	89	62.24

**Table 4:** Difficulties in dealing with depression symptoms among 78 nurses with depression working in the English-speaking regions of Cameroon from January – June 2018

Difficulties in dealing with symptoms of depression	Freq.	Percent
Not difficult at all	34	31.48
Somewhat difficult	60	55.56
Very difficult	9	8.33
Extremely difficult	5	4.63

### Predictors of depression

Significant predictors of depression on univariable analysis included: number of night shifts a week; total OBLI score; age; and the majority of shifts being night shifts (Tables 5 and 6). Recreational drug use perfectly predicted depression in this population.

**Table 5:** Univariable analysis for potential categorical predictors of depression among 143 nurses in the English-speaking regions of Cameroon assessed for depression from January – June 2018

Variable	Category	Total	N	%	OR	95% CI	*P* value
Gender (*n* = 143)	Male	47	28	59.57	1.18	0.54, 2.56	0.646
	Female	96	61	63.54			
Marital status (*n* = 142)	Single	73	50	68.49	0.60	0.28, 1.24	0.140
	Married	69	39	56.52			
Personal relationship (*n* = 127) ^a^	Yes	78	51	65.38	1.00	0.44, 2.26	0.993
	No	49	32	65.31			
Difficulties in personal relationship (*n* = 118)	Yes	26	19	73.08	1.75	0.62, 5.40	0.253
	No	92	56	60.87			
Monthly income sufficient (*n* = 133)	Yes	13	8	61.54	0.96	0.26, 3.97	0.946
	No	120	75	62.5			
Majority of shifts (*n* = 129)	Night	30	24	80.00	3.07	1.09, 9.93	0.021
	Day	99	56	56.57			
Regret career choice (*n* = 136)	Yes	23	18	78.26	2.66	0.87, 9.75	0.063
	No	113	65	57.52			
Life changing crises (*n* = 139) ^b^	Yes	57	34	59.65	0.85	0.40, 1.81	0.653
	No	82	52	63.41			
Presence of chronic illness (*n* = 140)^c^	Yes	17	12	70.59	1.54	0.47, 5.91	0.444
	No	123	75	60.98			
Alcohol consumption (*n* = 141)	Yes	71	46	64.79	1.23	0.59, 2.57	0.557
	No	70	42	60.00			
Recreational drug use (*n* = 142) ^d^	Yes	7	7	100.00			0.034
	No	135	81	60.00			
Hospital (*n* = 132)	Private	42	27	64.29	1.20	0.53, 2.78	0.638
	State-owned	90	54	60.00			

^a^Personal relationship was defined as close connections between two people formed by emotional and sexual interactions; ^b^ Life changing crises defined as loss of a loved one, physical or sexual trauma and condition of emotional or social instability ^c^ Chronic illnesses included: Asthma, chronic pelvic pain, diabetes mellitus, gastroesophageal reflux disease, chronic peptic ulcer disease, migraines, cerebral lesions and paralysis; ^d^ recreational drugs included: marijuana and tramadol

**Table 6:** Univariable analysis for potential continuous predictors of depression among 143 nurses working in the English-speaking regions of Cameroon assessed for depression from January – June 2018

Variable		Depression			No depression			*P* value
	Total (N)	n	Mean	SD	n	Mean	SD	
Age	108	66	28.35	5.16	42	31.95	7.85	0.005
Monthly income in USD	58	33	125.27	71.16	25	125.00	83.00	0.989
Number of children	105	62	1.77	1.56	43	2.00	1.81	0.497
Average weekly hours spent at work	127	78	10.02	4.48	49	13.86	16.29	0.051
Number of night shifts a week	101	58	2.50	1.68	43	1.77	1.25	0.018
Total OBLI score	143	89	40.11	5.42	54	35.48	4.91	<0.01
Quantity of alcohol consumed a week	41	24	2.06	1.59	17	1.49	0.75	0.178

*USD* United states dollars, *GPA* Cumulative grade point average, *OLBI* Oldenburg burnout inventory; Quantity of alcohol measured in units of alcohol consumed a week

**Fig. 1:**
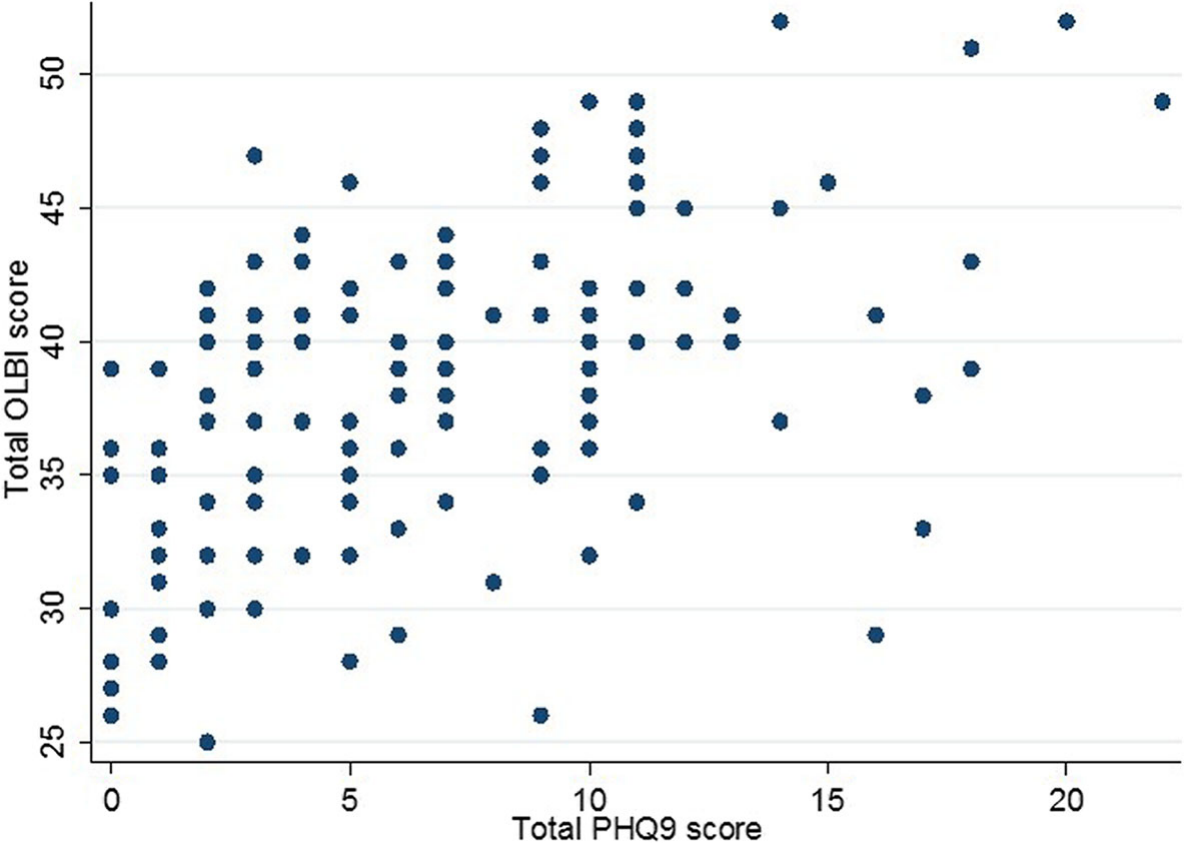
Scatter plot showing relationship between the total PHQ-9 score on the y-axis and the total OLBI score on the x-axis

After inputting the above listed significant predictors in a multivariable logistic regression model; Number of night shifts a week (aOR;1.58, *p* value; 0.045, 95%CI; 1.01, 2.48) and Total OLBI score (aOR;1.21, p value; 0.001, 95%CI; 1.08, 1.35) (Fig. 1) were found to be independent predictors of depression amongst the nurses recruited for the study (Table 7).

**Table 7:** Multivariable logistic regression analysis for independent predictors of depression among 143 nurses working in the English-speaking regions of Cameroon assessed for depression from January – June 2018

Variables	Adjusted odds ratio	*P* value	95% confidence interval
Majority of shifts (Night/Day)	2.15	0.316	0.48, 9.66
Age	0.94	0.195	0.86, 1.03
Number of night shifts a week	1.58	0.045	1.01, 2.48
Total OLBI score	1.21	0.001	1.08, 1.35
Recreational drug use^a^	1.00		

*OLBI* Oldenburg burnout inventory; ^a^Recreational drug use perfectly predicted the outcome

## Discussion

Our study aimed at determining the predictors of depression amongst nurses working in both private and public medical institutions in the English-speaking regions of Cameroon. Following statistical analysis, independent predictors of depression in our study population were; Number of night shifts per week; the total OLBI score and recreational drug use.

Due to increasing social and economic demands many professionals running night shift systems find difficulties with coping with the associated financial strain. These individuals work mostly during the later hours of the day and at night, resulting in significant disruptions in social and family life [24]. The above considerable strain coupled with the disruption in the normal psychological circadian rhythms associated with night shifts [24] has been shown to have strong associations with mental health conditions and symptoms including: insomnia, fatigue and depression [25–28]. The result could be an eventual decrease in staff performance; job satisfaction; exhaustion and eventually burnout [29], which as pointed out by our statistical analysis, is a significant predictor of depression in our study sample.

Adjustment of shift hours so that staffs work shorter night shifts; shift scheduling tailored to the personal problems of individual staff and the allocation of appropriate sleeping hours and sufficient resting days following night shifts, are some of the solutions proposed by authors [26, 29]. Such solutions are however difficult to implement in a setting like ours where nurses have to work extra hours and shifts, and resume work after just a few days off, in order to fill the void created by the critical shortage of nursing personnel.

The nursing profession could be highly demanding and stressful, be it emotionally or physically. Higher workload, increased turnover, and extended working hours secondary to critical shortage in nursing staff in the country [15], coupled with poor financial remunerations could only go a long way to make the working environment even more stressful and much more difficult to cope with. The end result is a decrease in job satisfaction, gradual exhaustion and disengagement from work and consequently burnout [29]. A lot of debate has however been raised on which of either depression or burnout actually leads to the other, given the overlapping nature of symptoms between the two conditions. The most convincing evidence of burnout leading to depression so far is the study carried out by Hakanen et al on Finish dentist students [30]. The cross-sectional design of our study however meant that we could not establish the direction of this relationship in our sample. This highlights the need for longitudinal studies with greater sample sizes aimed at this purpose.

In this study, recreational drug use was found to perfectly predict the outcome (depression). All seven participants who admitted to using recreational drugs, had depression. Nursing in Cameroon is quite stressful as nurses have to attend to numerous patients a day due to the low nurse-patient density ration in Cameroon. These nurses have to also deal with the emotional pressure of looking after critically ill patients daily. This could lead to the use of recreational drugs as an escape mechanism from these pressures.

The management of depression (resulting from working too many night shifts or from burnout syndrome) centers around the need for professional care and support. However mental health is highly neglected in Cameroon as is the case in most African countries [31, 32]. Consequently, mental health professionals and mental health institutions are far and wide apart, making it difficult for affected individuals to seek the professional help they need. This highlights the increasing need for the development and deployment of mental health institutions and professionals respectively around the country, in an effort to pay more attention to the mental health of Cameroonians in general, and high-risk individuals such as nurses in particular.

### Strengths and limitations

To the best of our knowledge, this was the first multicenter study to assess the prevalence and potential predictors of depression amongst nurses in Cameroon.

Due to the cross-sectional nature of the study, there is the potential for recall bias in the responses of the participants. Some of the items enquired about practices that are considered societal taboos (recreational drug use for example). This may have affected the way some participants responded to the questions.

Furthermore, the overlapping of symptoms between burnout and depression, coupled to the cross-sectional nature of the study design, made it difficult to assess temporality between the two constructs. Associations obtained should be interpreted with caution.

Findings obtained from our study could hardly be generalizable to the entire nursing population of the country as a whole or the English-speaking regions of the country in particular. This in part due to the convenience nature of our sampling and to the small nature of the sample size. An ongoing socio-political crisis in the two English speaking regions of the country at the time the study was carried out led to the exodus of individuals (nurses inclusive) from the region thereby reducing our sampling pool, and equally made movement to the various recruiting sites challenging.

## Conclusion

Depression is highly prevalent among nurses working in the English-speaking region of Cameroon. Use of accurate predictors could prove vital in the early detection and management of affected individuals especially in high risk professions like nursing. Predictors presented herein however require further investigation via multicentric nationwide studies, to obtain much more generalizable results.

## Data Availability

Not applicable.

## Abbreviations

OLBI: Oldenburg burnout inventory
PHQ-9: Patient Health Questionnaire-9
